# Vertebra Plana: A Narrative Clinical and Imaging Overview among Possible Differential Diagnoses

**DOI:** 10.3390/diagnostics13081438

**Published:** 2023-04-17

**Authors:** Andrea Angelini, Nicolò Mosele, Andrea Gnassi, Riccardo Baracco, Maria Grazia Rodà, Mariachiara Cerchiaro, Pietro Ruggieri

**Affiliations:** Department of Orthopedics and Orthopedic Oncology, University of Padova, 35128 Padova, Italy

**Keywords:** differential diagnosis, review, eosinophilic granuloma, fracture, spine, multiple myeloma, mnemonic

## Abstract

Vertebra plana is a rare radiologic condition characterized by a uniform loss of height of a vertebral body that represents a diagnostic challenge for surgeons. The purpose of this study was to review all possible differential diagnoses that may present with a vertebra plana (VP) described in the current literature. For that purpose, we performed a narrative literature review in compliance with the Preferred Reporting Items for Systematic Reviews and Meta-Analyses guidelines, analyzing 602 articles. Patient demographics, clinical presentation, imaging characteristics and diagnoses were investigated. VP is not a pathognomonic feature of Langerhans cell histiocytosis, but other oncologic and non-oncologic conditions should be considered. The list of differential diagnoses, based on our literature review, can be recalled with the mnemonic HEIGHT OF HOMO: H—Histiocytosis; E—Ewing’s sarcoma; I—Infection; G—Giant cell tumor; H—Hematologic neoplasms; T—Tuberculosis; O—Osteogenesis imperfecta; F—Fracture; H—Hemangioma; O—Osteoblastoma; M—Metastasis; O—Osteomyelitis, chronic.

## 1. Introduction

“Vertebra plana” (VP) is a form of severe, compression-induced vertebral collapse observed in a plain radiograph of the spine in lateral view. It appears as a uniform, somatic flattening of the complete vertebra, except the posterior elements and vertebral arch. Calvè was the first in 1925 to describe VP in a patient with spinal tuberculosis, with the radiographic definition of the “extreme collapse of a vertebral body with sparing of the posterior elements and normal to slightly widened adjacent disk spaces.” [1]. The term ‘‘vertebra plana’’ to define this condition was suggested by Buchman in 1927 [2]. However, there is not a universally accepted definition of VP in literature, including (1) a reduction of the anterior vertebral height of more than 70%, (2) a vertebral body collapsing to less than one-third of its original height, (3) the complete collapse of the anterior vertebral body with a minimal collapse of the posterior wall, creating a ‘pancake’-like flattening of the vertebral body, or (4) the symmetric flattening of the vertebral body [3,4,5]. These measurements have been described in both radiographs and mid-sagittal CT reformats, comparing prior imaging for a percentile calculation. Garg et al. [6] proposed a classification of VP in eosinophilic granuloma into Type 1 or Type 2, depending on the degree of vertebra collapse being less or greater than a 50% loss of height, respectively. Moreover, Type A lesions reflected a symmetrical loss of height, and Type B represented an asymmetrical loss of height.

Our approach for the aim of this study is clinically based on helping physicians approach patients with VP and unknown diagnoses rather than patients with already established diagnoses. VP is commonly known to manifest in the spinal involvement of Langerhans cell histiocytosis (LCH) or eosinophilic granuloma (EO) and over time has been linked to the disease as a pathognomonic finding [7,8,9,10], but may be caused by osteoporotic fractures in the adult population and other metabolic diseases [11,12,13], or different tumors [14,15,16]. However, investigation criteria among the existing studies are vastly heterogeneous and differ regarding the chances of promptly reaching a diagnosis and whether a biopsy could be helpful because other entities are frequently misdiagnosed or not considered in the differential diagnosis. The purpose of this study was to review all possible differential diagnoses that may present with VP as described in the current literature.

## 2. Focus on the Literature and Search Strategy

**Figure 1:**
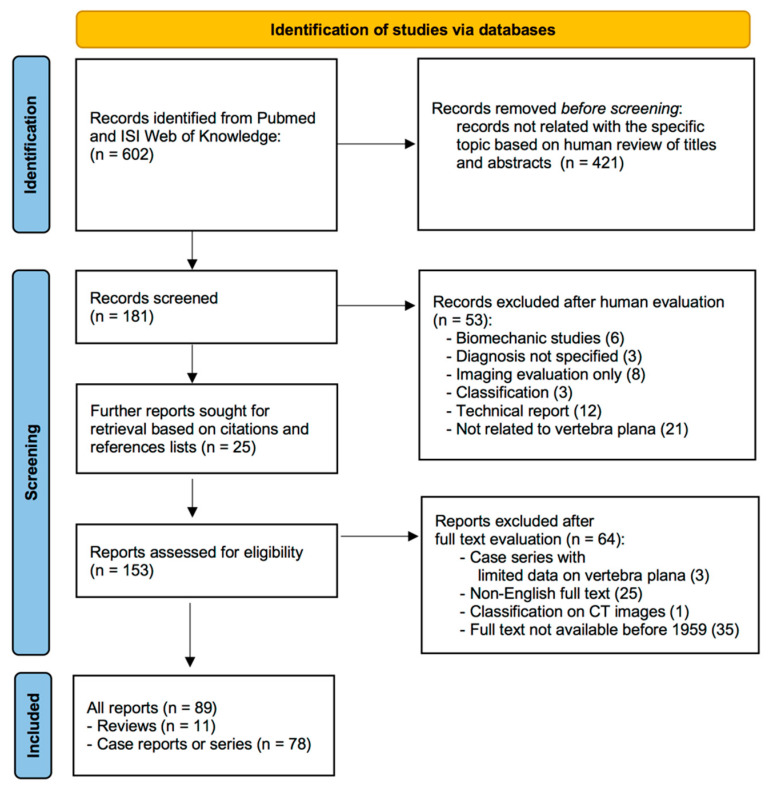
PRISMA flow diagram for reviews, which included searches of databases.

A search of the literature was done to identify studies reporting on VP, a series of patients with specific diagnoses associated with spinal involvement or patients treated for massive vertebral body collapse. English and non-English language literature were searched in Pubmed using the following terms in different combinations and in the ISI Web of Knowledge database: “spine”[MeSH Terms], “spine”[All Fields], “vertebra plana,” “Langerhans cell histiocytosis,” “Calve’s disease,” “vertebral body compression fractures,” “kummel disease,” and “flat.” The definitive search was done using the following: “spine”[MeSH Terms] AND (“vertebra plana “OR” Langerhans cell histiocytosis” OR “Calve’s disease” OR “vertebral body compression fractures” OR “kummel disease” OR “flat” NOT “flat back”), resulting in 602 results from 1946 to date (mainly small scaled or consist of case series and case reports). Moreover, we improved the search strategies by looking at the citations and reference lists of the main papers on the topic. The search was carefully revised according to PRISMA guidelines [17], and the eligibility criteria of included studies have been summarized in a PRISMA flow diagram (Figure 1).

## 3. Differential Diagnosis within Oncologic Diseases

### Langerhans Cell Histiocytosis

One of the most frequent causes of VP is LCH, a disease characterized by the uncontrolled pathologic proliferation of Langerhans-like dendritic cells. This disease can affect various organs such as bones, liver, skin or lungs and entire apparatus such as the endocrine system. LCH is currently classified into two groups based on the involvement of organs and sites: the first is SS-LCH (single system), and the second is MS-LCH (multisystem). In SS-LCH, only one organ or system is involved, and in the case of bone, it was previously known as eosinophilic granuloma [7]. Incidence of LCH in the population ranges between 1:1,500,000 and 1:2,000,000 [18], and most of the cases have been reported in childhood, with a peak (nearly 80% of the cases) in children between 5 and 10 years [10,19,20].

**Figure 2:**
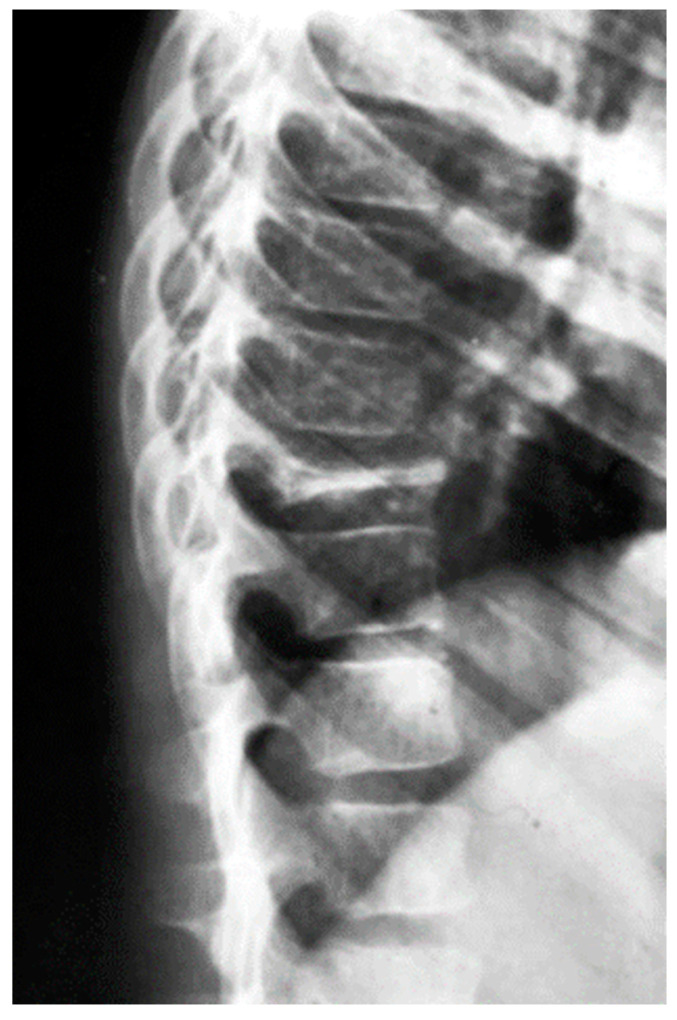
Lateral thoracic spine radiographs of a 7-year-old boy presenting with atraumatic back pain, with vertebra plana diagnosed as Langerhans cell histiocytosis (eosinophilic granuloma).

In bone SS-LCH, the most frequent localizations are the skull, ribs, pelvis, or the metaphysis of long bones [21,22,23,24]. Spinal localization has been reported to be around 7–25% [10,25,26,27,28,29]. The most common spinal localization is the thoracolumbar tract (Figure 2), but several cases of cervical spine involvement have been reported both in adults and in children [29,30]. The etiopathogenesis of this disease is still unknown, but several hypotheses have been made (immunological or genetic dysfunction and external agents such as the Epstein–Barr virus or bacteria) [31,32,33,34,35].

**Figure 3:**
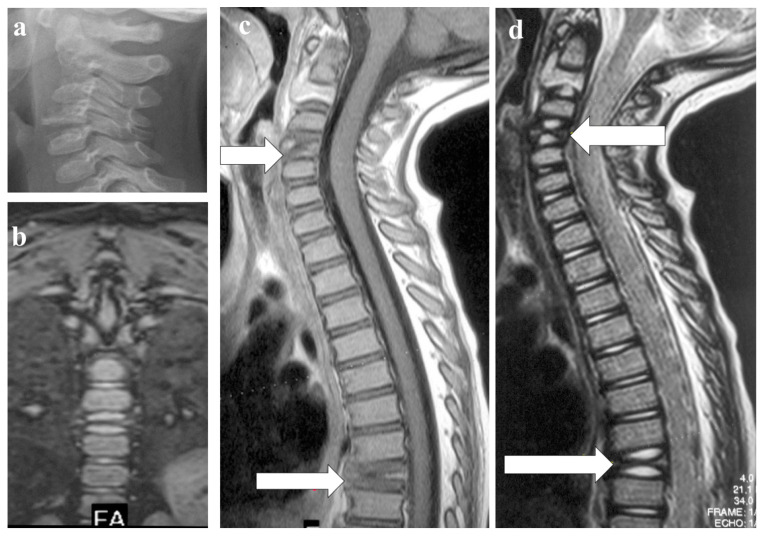
Langerhans cell histiocytosis in a 24-year-old male. (**a**) Cervical X-ray in lateral view shows vertebra plana in C4. (**b**) MRI of the cervical and thoracic spine demonstrates further vertebra plana in T8. Both vertebrae present flattening of the anterior body (with arrows) without compression of the spinal cord. (**c**) T1 sequence. (**d**) T2 sequence.

Radiological findings depend on the stage of the disease: initially, it can be seen just as a lytic lesion in vertebral bodies without progressive growth, but sometimes it can lead to a major vertebral collapse causing VP [8,9,22,32]. MRI with contrast is usually the second stage imaging preferred for this type of pathology since it allows to see also soft tissue involvement, which, in the case of vertebral lesions, can be present in nearly half of the cases [30]. Sometimes multiple lesions may be observed (Figure 3).

**Figure 4:**
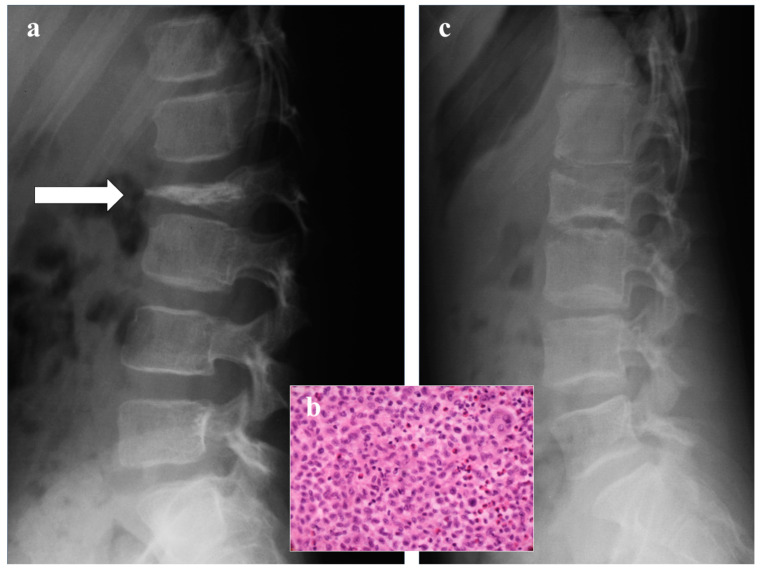
Langerhans cell histiocytosis (eosinophilic granuloma) in a 14-year-old male. (**a**) Lumbosacral radiograph shows vertebra plana in L2. (**b**) trocar biopsy and histologic evaluation (high power hematoxylin and eosin view—original magnification ×40) confirmed the diagnosis: a clonal proliferation of cells that morphologically and immunophenotypically resemble Langerhans cells (**c**) complete healing and restoration of the vertebral body at 6 years after intralesional corticosteroid injection.

Diagnosis can be quite challenging since it has to be distinguished from all the other lytic lesions that can affect vertebral bodies, such as multiple myeloma, Ewing sarcoma or tuberculosis and all the other infectious diseases. The only method to obtain a diagnosis is a biopsy (Figure 4) [30,32,36,37,38,39]. The accuracy of the diagnosis raises when the biopsy is performed under a CT guide [39,40,41,42,43].

## 4. Ewing’s Sarcoma

VP can also rarely be caused by Ewing’s sarcoma (ES). Ewing’s family tumors are a group of highly malignant small round-cell neoplasms of neuroectodermal derivation that affect mainly the younger population, with a peak between 10 and 20 years. ES is the second most frequent malignant osseous tumor after osteosarcoma in children [44].

Primary localization in the spine is quite rare, with posterior vertebral elements usually affected first. In some cases, the disease extends to the vertebral body and may cause a collapse. Sacral localization is generally considered apart from the spine. This division has been based on the differences in the radiotherapy treatment responses where radiotherapy-treated ES has been seen to have a better prognosis in non-sacral localization [45]. Nowadays, less than 10 cases of ES causing VP have been reported in the literature [14,15,46,47,48,49,50]. Two cases of ES were reported in 1975 by Poulsen et al. on a 2-year-old with L4 involvement and a 9-year-old girl with L5 localization [14]. Reinius et al. in 1984 reported six cases in the intergroup Ewing’s Sarcoma Study (IESS) [46], and then only 6 more cases were reported: Kager et al. described an 8-year-old girl with a C3 localization [49], and another was reported by O’Donnell et al. [50]; the case of a 7-year-old child with a T11 involvement was reported in 2002 by Papagelopulos et al. [15], a 13-year-old boy with an L4 ES causing VP was reported in a letter to the editor in 1999 [47] and more recently Patnaik et al. reported their experience in uncommon localization of ES reporting two cases of VP in their experience [51].

## 5. Hematologic Neoplasms

**Figure 5:**
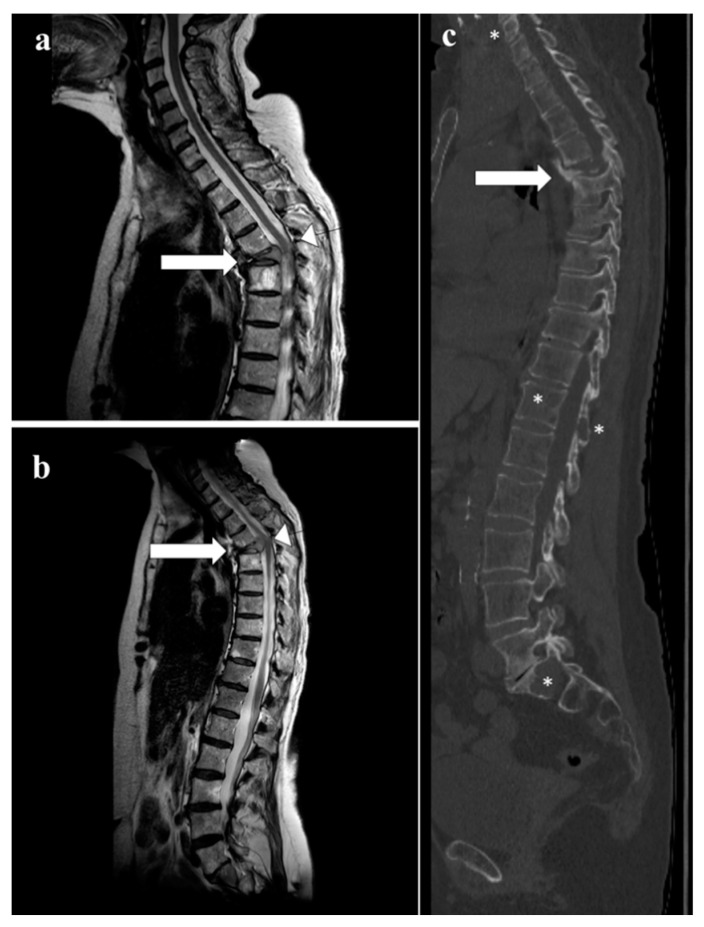
Multiple myeloma in a 74-year-old female, with pathological fracture of T5 vertebra and medullary compression as the first symptom. T2-weighted sagittal MRI of the cervicothoracic (**a**) and lumbar spine (**b**) showing an approximately 90% loss of vertebral height at T5 (white arrow) with canal compromise (small head-arrow). (**c**) Sagittal CT scan confirming the pathological fracture (white arrow) and multiple osteolytic lesions (white asterisks).

Among the rarest causes of VP, we can also include localization from hematologic neoplasms such as leukemia [52], lymphoma [53,54,55] and myeloma [56,57]. VP has been reported to be the first presentation of spinal-localized Non-Hodgkin lymphoma (NHL) in rare cases; in a case report, it was shown to lead to paraplegia [55]. It remains clearly a rare presentation since the primary spinal localization of NHL is very rare, and VP has been seen in only a few cases. Obviously, NHL has a different target of patients compared with LCH. Indeed, the prevalence of this disease is much higher in the sixth or seventh decade of life [55]. Leukemia is another rare cause of VP. Baky et al., in 2020, reported two cases of leukemia in pediatric patients. In pediatric cases, the most frequent kind of leukemia causing VP is acute lymphoblastic leukemia, and in these cases, a bone marrow biopsy has been performed to confirm the diagnosis [16]. Moreover, multiple myeloma (MM), which is the most frequent type of primary bone malignant tumor, can be another rare cause of VP (Figure 5).

**Figure 6:**
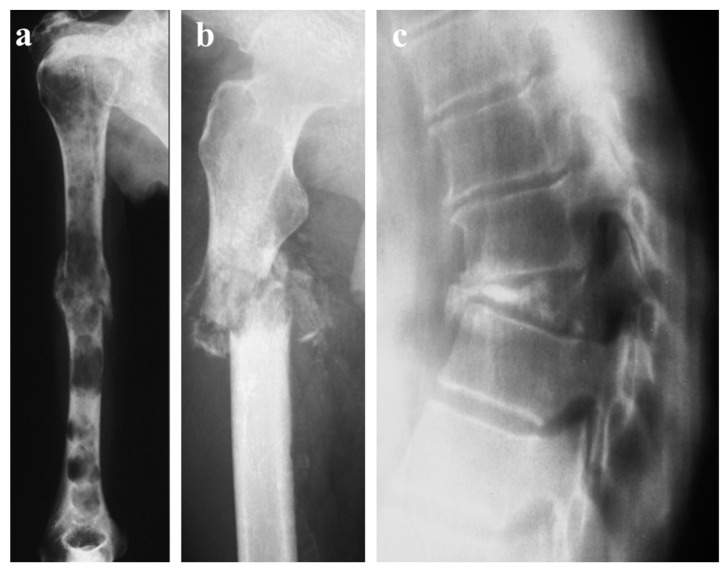
Multiple myeloma with aggressive behavior and multiple pathological fractures at initial presentation. (**a**) involvement of the humerus, (**b**) pathological fracture of the femoral shaft and (**c**) vertebra plana at the T8 level.

Greenleaf et al. described an adolescent patient with VP as the initial presentation of a MM [56], whereas VP is usually observed during follow-up or in diffuse bone involvement (Figure 6) [58,59,60,61].

## 6. Bone Metastases

**Figure 7:**
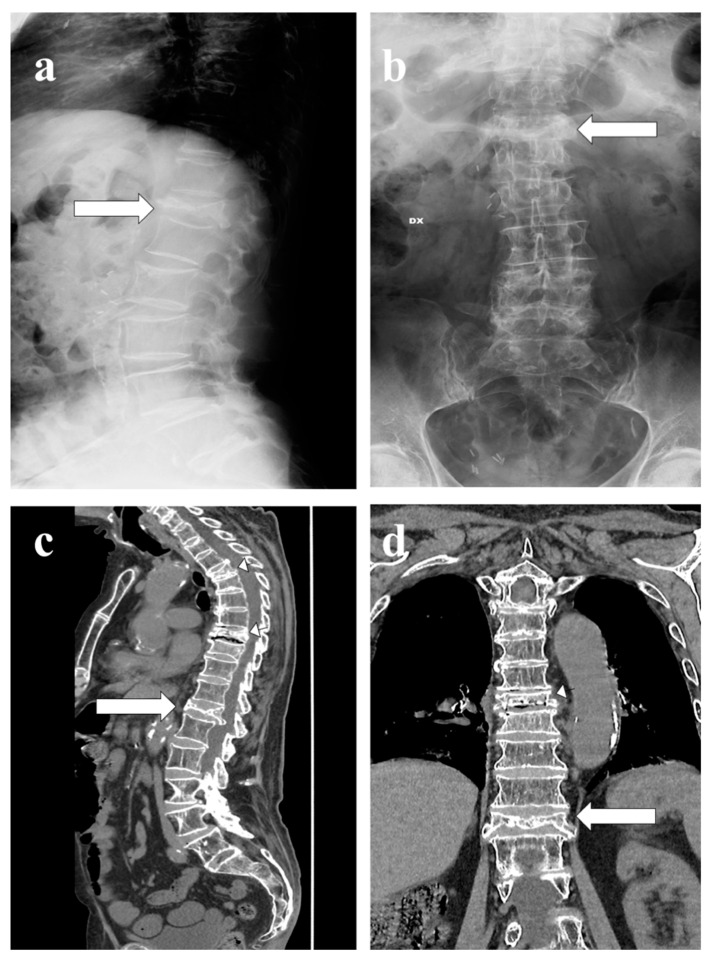
Vertebra plana in metastatic carcinoma. An 87-year-old female was treated 2 years before with resection of the sigma for colorectal carcinoma. (**a**) lateral and (**b**) anteroposterior radiographs show clear vertebra plana at the T12-level (white arrow). CT scan in (**c**) sagittal and (**d**) coronal view show multiple pathologic fractures (arrowheads) in the thoracic spine.

Obviously, each osteolytic bone metastasis can lead to the severe compression of vertebral bodies (Figure 7), but only a few studies have been conducted about this kind of etiology.

**Figure 8:**
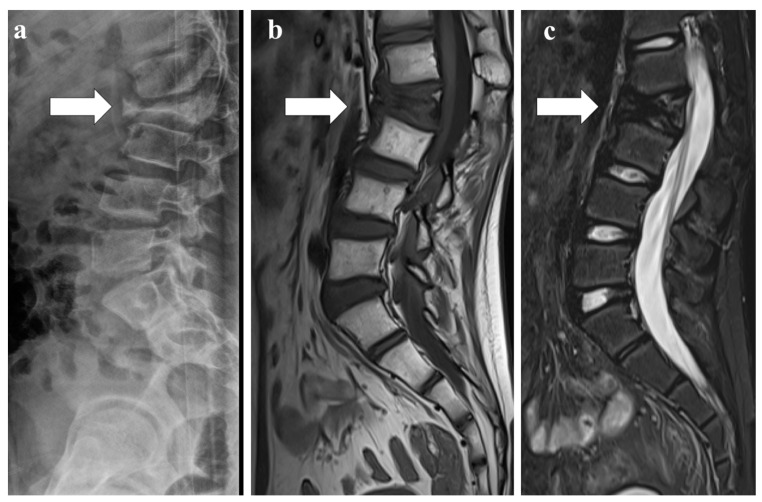
Bone metastasis from osteoblastic osteosarcoma of the extremities in a 21-year-old male. (**a**) lateral radiograph of the lumbar spine shows a vertebra plana in L1 with local kyphosis (white arrow). (**b**) sagittal enhanced T1 and (**c**) sagittal T2 STIR MRI confirmed the fracture with approximately 80% collapse and no signs of dural compression.

Hentschel et al. reported the results of percutaneous vertebroplasty in vertebra plana secondary to metastatic thymic carcinoma [62], such as other papers on metastatic diseases that are more focused on palliative treatments than etiological causes of VP [63]. Rarely, also osteosarcoma or Ewing’s sarcoma may metastasize to the spine, causing vertebral collapse (Figure 8).

## 7. Osteoblastoma

Another recognized rare cause of VP can be osteoblastoma (OB). Spinal localization of OB is common, attesting between 32% and 45% [64]. Usually, the vertebral localization is limited to the posterior elements, but in more aggressive cases, it can affect anterior vertebral bodies as well, causing compression fractures that can radiographically appear as VP. Based on our literature review, only two cases of OB appearing as VP have been reported; in both cases, the lesions were localized in the thoracic tract [65,66].

## 8. Giant Cell Tumor of Bone

Another neoplastic pathology that can cause vertebral body collapse is giant cell tumors (GCTs) of the bone. These are commonly considered benign tumors but can be locally aggressive and give lung metastases. It has a major incidence in the third and fourth decade of life [67], with a slight predominance of females compared to males. Vertebral localization is not typical since it is localized more often in the meta-epiphyses of the long bones; in fact, only 2.7% of GCTs affect the spine [68]. Spinal localization occurs more often in the sacrum [69,70], but it can also be found in the thoracic, lumbar [16] and cervical vertebrae [71,72]. The mass can also compress the spinal cord, producing neurological symptoms [73]. Vertebral localization is mainly in the vertebral body, but it can also involve posterior elements, especially in more aggressive cases [67].

## 9. Myofibromatosis

Besides LCH, another cause of VP in childhood may be myofibromatosis. This is a rare congenital disease with an incidence of around 1:400,000 in newborns that can appear with well-vascularized subcutaneous nodules [74,75]. These lesions more frequently affect a single site (especially subcutaneous tissues in the upper body), but in nearly 40% of cases, the multicentric form has been reported. The multicentric form can involve the gastrointestinal system, the bones and, in some cases, the central nervous and cardiopulmonary systems. In 1995 the first case of VP in a child suffering from myofibromatosis was reported [76], and five other cases have been reported afterward [77,78,79].

## 10. Rare Entities: Aneurysmal Bone Cyst, Hemangioma, Primary Germ Cell Tumor

In 2006, Codd et al. reported a pediatric case of VP caused by an aneurysmal bone cyst (ABC) [80]. A diagnosis of ABC related to VP is very rare, and only two more cases have been reported in the literature to the best of our knowledge [81,82]. One case of hemangioma causing VP has been reported: in the beginning, the diagnosis was uncertain since it occurred in a patient with a diagnosis of pineal and suprasellar germinoma, but after performing a biopsy, it was diagnosed as hemangioma and not a metastasis [83]. In 2020, a case of VP secondary to a primary germ cell tumor has been described, but this is the only case reported in the literature, as well as sacral teratoma and rhabdomyosarcoma, which has been seen to cause VP [16].

## 11. Differential Diagnosis within Non-Oncologic Diseases

### Infections

Some bacterial infections have been correlated with VP. Tay and Wong reported a case of VP (T5 vertebra) caused by Nocardia Kroppenstedtii in a 78-year-old female on long-term immunosuppressive therapy with steroids for underlying autoimmune hemolytic anemia [84]. They found vertebral body collapse and erosion of superior and inferior margins at MRI, associated with bilateral limb weakness, the elevation of C-reactive protein and high white cell counts. Diagnoses have been made using the 16S rRNA gene sequencing approach on pus samples drained from the T5 abscess. Other cases of these saprophytic gram-positive aerobic actinomycetes (or other variants such as N. Asteroides) that can lead to life-threatening multisystemic infections in immunosuppressed patients have been reported in the literature [85]. VP has also been observed in rare cases of disseminated coccidioidomycosis [86,87].

Tuberculosis (TBC) with bone involvement most commonly affects the lower thoracic region in younger patients and the upper lumbar region in elder patients. The first description of VP correlated to tuberculosis goes back to the 80′s [88]. Bone involvement is one of the most common extrapulmonary locations of TBC that is present in almost 25% of tuberculosis infections; of these, 11% are osteoarticular [89,90]. Sureca et al. studied a group of 451 children in India from 2001 to 2010, observing 2.44% of sick people with TBC affecting bones; vertebral lesions are not usually isolated, but different bones are affected at the same time [91]. Studies on MRI of these patients confirmed that in the majority of these cases, the lesions are noncontiguous [92]. In 2015, Haghighatkhah et al. reported a case of a 5-year-old female with VP and multiple lytic lesions highly suspicious for LCH. However, further evaluations, including histopathologic analysis, confirmed an infection from Mycobacterium tuberculosis, and the patient was healed after treatment with anti-tuberculosis drugs [93]. In adults, the formation of abscesses can result in vertebral body destruction and, when the involvement of the pedicles occurs, it usually is associated with severe vertebral body collapse [94]. Clearly, the presence of caseating granuloma on histological examination suggests TBC and microbiological confirmation is essential for a definitive diagnosis.

## 12. Chronic Recurrent Multifocal Osteomyelitis

Chronic recurrent multifocal osteomyelitis (CRMO) is a chronic autoinflammatory disorder due to infective or non-infective etiologies, rarely reported in the spine as a primary presentation [16,95,96,97]. Baky et al. reported two patients with CRMO initially presenting with vertebral collapse [16], as has been previously reported in three out of seven children who had CRMO [98]. All authors strongly recommended biopsy for diagnosis, and the results of cultures must be negative before therapy with antibiotics can be withheld [16,98]. In a study of whole-body MRI of patients with CRMO, 35% of patients had spinal lesions, and two of these were VP [99]. Clearly, diagnosis of CRMO should be considered when other differential diagnoses such as malignancies (leukemia, lymphoma, primary and secondary bone tumors), immunodeficiency (e.g., defects in the IL-12 interferon axis may be accompanied by mycobacterial infections), LCH or other disorders have been excluded. The non-infective etiology is not still clear; Hofman et al. reported a contribution of some cytokines involved in the chronic inflammatory activity as a causative mechanism of VP, eroding the superior and inferior parts of the bone [100].

## 13. Osteogenesis Imperfecta

Osteogenesis imperfecta is a rare genetic condition with abnormalities in the synthesis of collagen type I that, in some cases, can produce a severe form of scoliosis and vertebral deformities. VP has been rarely described between these abnormalities [16,101]. Baky et al. reported two cases of VP in newborns with a collapse <50%, one at the L2 vertebra diagnosed at ultrasound evaluation and another at both the L2- and T9-levels as incidental findings [16].

## 14. Fractures

**Figure 9:**
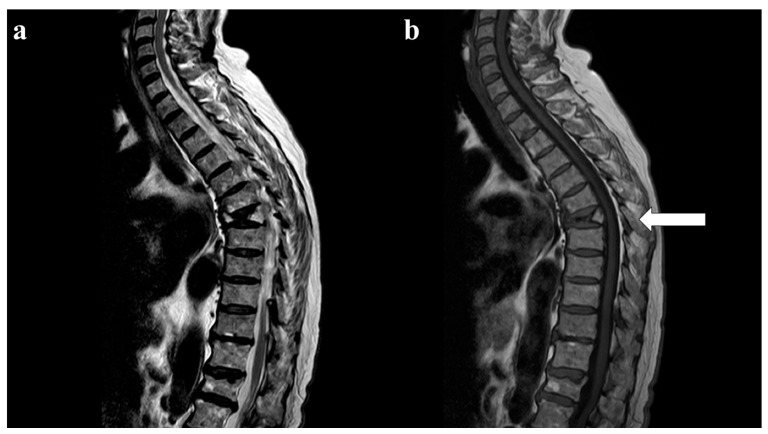
Post-traumatic vertebra plana in a 75-year-old female. (**a**) Sagittal T2 and (**b**) T1 weighted MRI show acute wedge fractures of T7 without the involvement of the posterior wall and medullary canal (white arrow).

Beyond the above-mentioned causes, we should consider traumatic etiologies where strengths act on the bone, causing a collapse (Figure 9), even in childhood [102,103].

**Figure 10:**
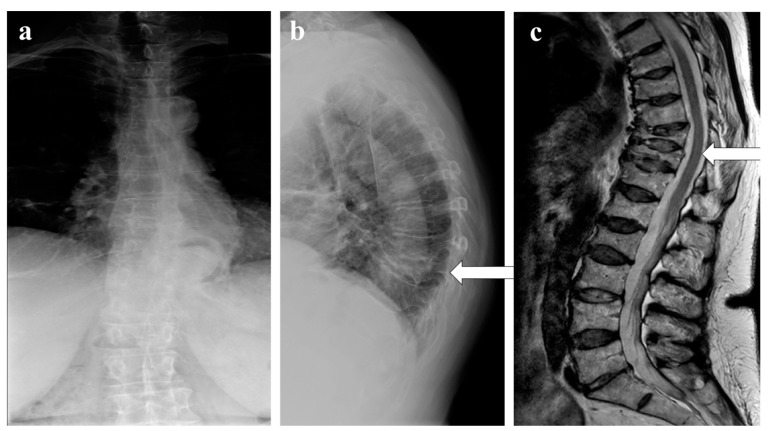
Osteoporosis and fragility fractures in an 85-year-old female. (**a**) anterior and (**b**) lateral radiographs show low bone mineral density and spinal deformity. Note the increase in the kyphotic angle on the thoracolumbar spine associated with vertebra plana (white arrow). (**c**) Sagittal T2-weighted MRI showing old vertebral wedge fractures, including T10 and T11, with a reduction of the anterior vertebral height of more than 70% (white arrow). Lack of edema on MRI indicating old fracture.

Low bone mineral density leading to fragility fractures is a highly prevalent yet underdiagnosed condition in the aging population (Figure 10). Osteoporosis is one of the major public health problems and is becoming increasingly prevalent in both men and women.

**Figure 11:**
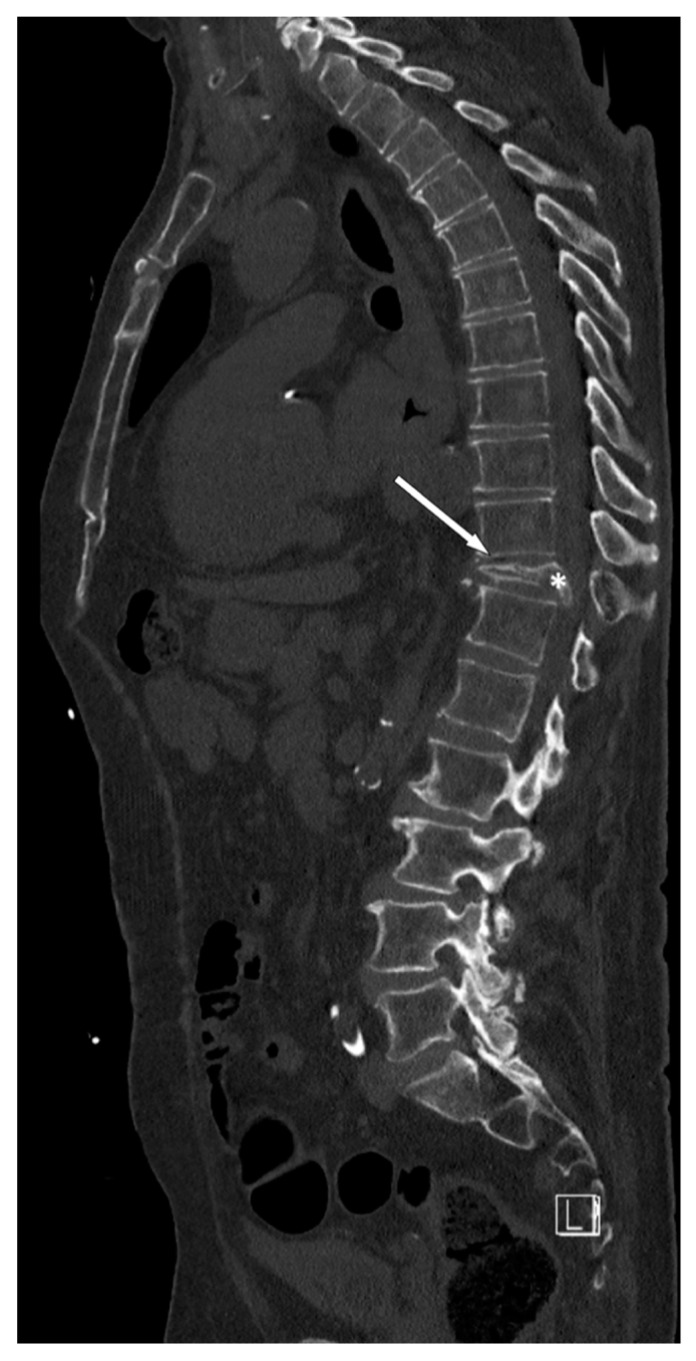
A 70-year-old male with amyotrophic lateral sclerosis was admitted to the emergency department due to progressive respiratory failure and hyponatremia. Sagittal CT scan showing vertebra plana in T11 with neurologic compression from vertebral body collapse (asterisk). A diagnosis of atraumatic chronic osteoporotic fracture is supported by disc degeneration suggested by vacuum disc (white arrow) and bone sclerosis.

There is also a wide range of pathological conditions and drugs that can cause a progressive bone mineral density reduction: diabetes mellitus, hyperparathyroidism, leukemias, gastroenterological diseases (intestinal malabsorption and cirrhosis), rheumatic disease and renal disease that can alter the bone’s metabolic balances (Figure 11). Some drugs, such as glucocorticoids, heparin, proton pump inhibitors and immunosuppressants, cause worse conditions in those already compromised [104].

**Figure 12:**
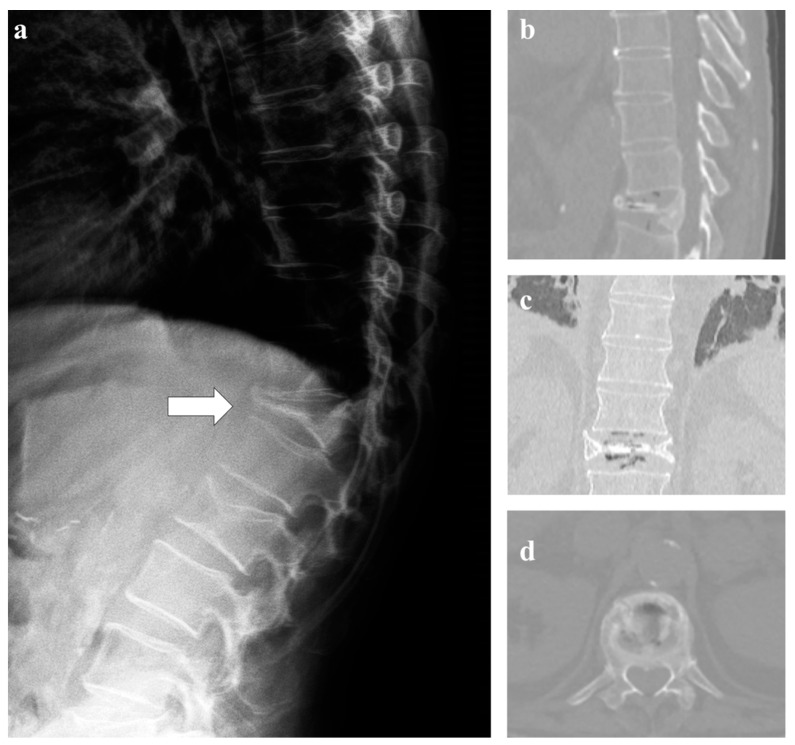
A 62-year-old male was admitted with severe backache after a long history of prednisolone therapy for rheumatoid arthritis. (**a**) A diagnosis of Kummel disease has been performed after a progressive collapse of the vertebra. Sagittal (**b**), coronal (**c**) and axial (**d**) CT scan shows intra-vertebral “vacuum” associated with air in the adjacent disc spaces, signs of symptomatic vertebra plana.

Kummel disease is the eponym for avascular necrosis of the vertebral body after a vertebral compression fracture. This is quite a rare complication and represents a failure of the fracture healing process [105]. Radiographically, the collapsed vertebra (typically lower thoracic and upper lumbar) presents an intravertebral vacuum cleft and fluid, accentuated on extension stress lateral views (Figure 12).

## 15. Principles of Treatment

The treatment of VP has been controversial for many years. Progressive spontaneous recovery of vertebral height has been occasionally observed in patients with LCH [106], with or without corticosteroid injection (Figure 4), and some authors suggest surgical treatments only in case of neurological impairments secondary to vertebral collapse [107]. Indications for the interventional approach are mainly based on the partial restoration of the somatic body height, the relative reduction of spinal kyphosis caused by the severe collapse and spinal stability. All these objectives can be achieved with multilevel pedicle screw fixation (open or percutaneous), percutaneous vertebroplasty or balloon kyphoplasty [108,109,110]. To obtain firm stabilization, both anterior and posterior column support is usually required [11,105]. The major bone compactness in the posterior body wall of collapsed vertebra seems to be a favorable prognostic factor for good results of percutaneous vertebroplasty [12]. However, some aspects should be considered, such as the underlying bone stock, the global kyphosis and local kyphotic deformity at the VP and the patient’s comorbidities. Obviously, the treatment of the VP should be combined with chemotherapy, radiation or surgery of the primitive tumor in oncologic patients.

On the other hand, the relative or absolute contraindications for interventional therapies are technical difficulties with the percutaneous approach [13,106,107,108,109,110,111,112,113,114]. Recent studies reported that vertebroplasty could be no more a contraindication thanks to the radio-guided approach and the reduced risk of medullar lesions or cement leakage into the spinal canal or into the perivertebral veins [12,115,116]. Others demonstrated the efficacy of third-generation vertebroplasty, percutaneous kyphoplasty with an expandable SpineJack implant, reporting a correct and stable reconstruction of the vertebra [115,117].

**Limitations of the study.**

The first limitation of the study is related to the intrinsic subjective nature of “narrative reviews,” which are useful for obtaining a broad perspective on a specific topic but with possible authors’ bias on study selection. The possibility of drawing misleading conclusions is based on selection bias, subjective weighing of the studies chosen for the review and unspecified inclusion criteria. In fact, we included the criteria used for the accurate literature review according to the PRISMA guidelines, with the determination of included studies, the way they are analyzed, and the conclusions drawn.

The second limitation is based on the difficulty in determining complex interactions between a large set of studies involved with different methodological aspects. Only a few articles focus on the topic of VP, so we have included and evaluated case series grouped by procedure type, tumor histology, or imaging characteristics.

**Focus on take-home messages.**

A number of conditions can lead to the complete collapse and flattening of an isolated vertebral body, with imaging and clinical features of a VP. The management of these patients and the teamwork approach is significantly different based on the underlying cause. Furthermore, as in our clinical experience, the finding of a VP is not uncommon, and the physician should be advised of the widest range of differential diagnoses.

## 16. Conclusions

**Figure 13:**
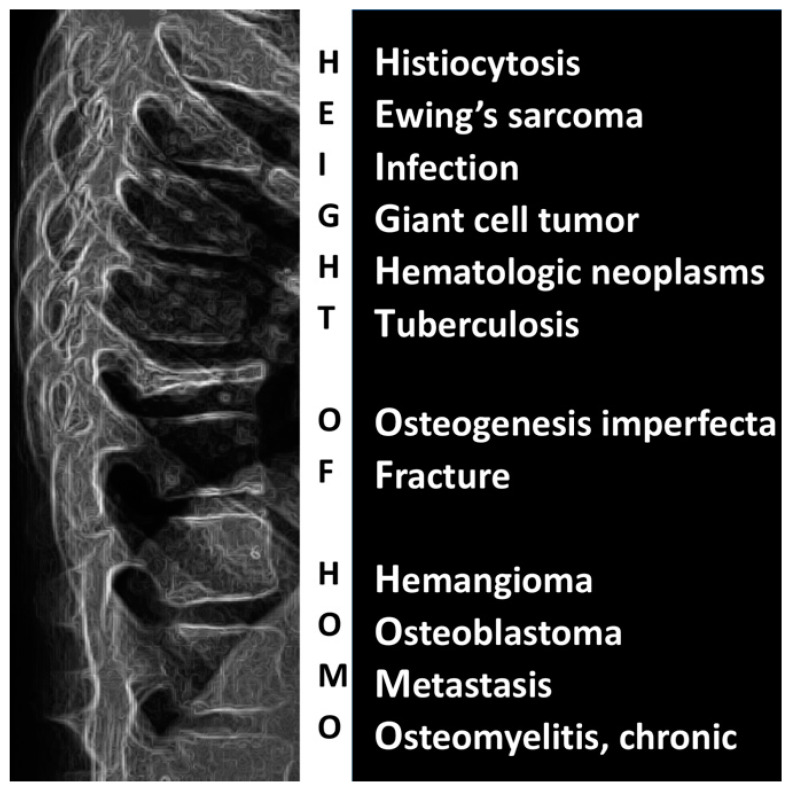
The mnemonic HEIGHT OF HOMO summarizes all possible differential diagnoses for vertebra plana.

Detailed data in the current literature on the presentation and etiopathogenesis of VP are scarce and lack structure, yet some characteristics can be found. Few papers focused on the topic, whereas most of the existing are vastly heterogeneous and differ regarding the strategy to reach the diagnosis. First of all, the definition of VP is not unique, creating a selection and evaluation bias in the literature. We strongly suggest the use of a unique definition of VP as follows: a severe (reduction > 70% of anterior vertebra height compared to adjacent cephalic level vertebra) compression-induced vertebral collapse, with somatic flattening and local kyphosis angle > 15°, evaluated in a plain radiograph of the spine in lateral view. Moreover, we would point to the need for a careful multisystem evaluation considering laboratory findings, history-taking, and physical examination, even because biopsies of VP present high percentages of non-diagnostic results. Incisional biopsy in the spine may lead to substantial morbidity and is not recommended as a first-line work-up procedure. We suggest the use of percutaneous trocar biopsy in the clinical suspicion of LCH, ES, hematologic neoplasms (excluding myeloma), infections and in all conditions in which the collapse of the vertebra cannot be clearly related to a traumatic event. Obviously, in the presence of neurological deficits requiring emergent decompression, intraoperative samples should be taken for both histologic analysis and cultures. VP can represent conditions other than eosinophilic granuloma, and the list of differential diagnoses, based on our literature review, can be recalled with the mnemonic HEIGHT OF HOMO: H—Histiocytosis; E—Ewing’s sarcoma; I—Infection; G—Giant cell tumor; H—Hematologic neoplasms; T—Tuberculosis; O—Osteogenesis imperfecta; F—Fracture; H—Hemangioma; O—Osteoblastoma; M—Metastasis; O—Osteomyelitis, chronic (Figure 13).

## 17. Essentials

- A clear, widely accepted radiographic definition of vertebra plana is mandatory;
- Vertebra plana should be defined as a severe (reduction > 70% of anterior vertebra height compared to adjacent cephalic level vertebra) compression-induced vertebral collapse, with somatic flattening and local kyphosis angle > 15°;
- Vertebra plana is not a pathognomonic feature of eosinophilic granuloma (Langerhans cell histiocytosis), but other conditions should be considered;
- Differential diagnosis in the setting of a vertebra plana includes tumors and non-oncologic disease, justifying a systematic approach in these patients.

## Data Availability

Manuscript data are embedded in the text and fully available on specific request.
